# Antibiotic and cytotoxic peptides

**DOI:** 10.3762/bjoc.8.127

**Published:** 2012-07-24

**Authors:** Norbert Sewald

**Affiliations:** 1 Bielefeld University, Department of Chemistry, Organic and Bioorganic Chemistry, Universitätsstr. 25, 33615 Bielefeld, Germany

**Keywords:** antibiotic, cytotoxic, peptides

Through evolution, nature has provided practically all organisms with secondary metabolites: chemical compounds that provide the producing species with specific protective mechanisms or other advantages. Among these, there are, e.g., peptides that display antibiotic/antimicrobial activity. These have been isolated from plants, invertebrates, vertebrates, and humans, but also from microorganisms. Some antibiotic peptides are an essential part of innate immunity, which has evolved in most organisms to combat microbial challenge. In addition, antimicrobial peptides sometimes also display cytotoxicity against mammalian cells.

There are several good reasons for a Thematic Series in the *Beilstein Journal of Organic Chemistry* on “Antibiotic and cytotoxic peptides”: The fight against bacterial infections is in a critical phase. The excessive application of antibiotics in human therapy, and also in livestock breeding, leads to the rapid development of highly virulent bacterial strains resistant to the conventionally applied antibiotics. Consequently, the search for new antibiotically active compounds is of premier importance. On the other hand, highly cytotoxic peptides and peptide analogues, such as monomethyl auristatin E, are used as the “warhead” in antibody–drug conjugates for tumor therapy, as approved recently by the FDA [1].

It is a privilege of synthetic chemists to be able to modify natural products by total synthesis. Many naturally occurring compounds are highly complex, and their efficient total synthesis often is out of reach. However, in frequent cases structural simplifications have proven amenable, and the resulting compounds have sometimes been shown to retain biological activity. The challenge of discovering new antibiotically active or cytotoxic compounds persists. Moreover, peptides have experienced a renaissance during recent years with respect to their application in Medicinal Chemistry [2].

This Thematic Series on “Antibiotic and cytotoxic peptides” presents contributions from synthetic chemists active in the fields of peptides, peptidomimetics, drug design, and method development, in order to promote the research area further and to disseminate the interdisciplinary research efforts to a broader audience.

Norbert Sewald

Bielefeld, July 2012
